# Influence of germination on the bioactivity, structural, functional and volatile characteristics of different chickpea flours

**DOI:** 10.1016/j.fochx.2024.101195

**Published:** 2024-02-13

**Authors:** Hongyan Mao, Shuo Yuan, Qin Li, Xiaoyan Zhao, Xiaowei Zhang, Hongkai Liu, Ming Yu, Meng Wang

**Affiliations:** a Research Institute of Grain Crops, Xinjiang Academy of Agricultural Sciences, Urumqi 830091, China; b Department of Food Science and Nutrition, Culinary Institute, University of Jinan, Jinan, Shandong 250022, China

**Keywords:** Germination, Chickpea, Bioactivity, Protein secondary structures, Functional characteristics, Volatile compounds

## Abstract

In this paper, the objective was to evaluated the impact of germination of chickpea cultivars (Muying1, Y2-514 and YZ-364) on the bioactivity, volatiles and functional properties. The results showed that the Vitamin C content of Muying1, Y2-514 and YZ-364 after germination significantly increased (*p* < 0.05). Moreover, the germination also caused a significant decrease in lower transition temperatures and enthalpy values in chickpea flours (*p* < 0.05). After germination treatment, β-sheet and random coils in protein secondary structures increased and β-turn decreased in YZ-364; α-helix, β-sheet and random coil in Y2-514 and Muying1 decreased, while β-turn increased. The germination significantly enhanced the functional properties of three chickpea flours (*p* < 0.05). It was proved that the germination significantly enhanced the total phenolic and flavonoids content, antioxidant activity and in vitro protein digestibility. The GC-IMS revealed that the germination could affect the contents of volatile compounds of chickpea flours.

## Highlights

• Germinated chickpeas had lower transition temperatures values and enthalpy values.
• The germination improved the WHC, OHC, EA, ES, FC of chickpea flours.
• Germinated chickpeas showed higher antioxidant activities.
• The conformation changes of protein during the chickpea seed germination.
• Some volatiles determined by GC-IMS increased in germinated chickpea volatiles.

## 1 Introduction

Chickpea (*Cicer arietinum L.*) is one of most important legume crops in the world, and accounts for 17 % of global legume production (FAO, 2020). Chickpea is divided into two main categories, which is desi and kabuli, respectively. Desi seed is smaller and edges with dark brown coat, while Kabuli seed is a larger and smoother with lighter colored coat (FAO, 2020). Chickpea also is a nutrient-rich pulse, which not only contains rich protein and carbohydrate, but also unsaturated fatty acid, dietary mineral, dietary fibres, nonstarch polysaccharide and isoflavones (Gupta, Sharma, Das, Ansari, & Dwivedi, 2017). In addition, chickpea has also been proven to have many health effects, such as preventing cancer, hyperlipidemia, nervous system development and cardiovascular diseases (Gupta et al., 2017), which has been widely used to develop functional food because of its bioactive components, outstanding and potential nutritional values for human.

In general, the pretreatment of chickpea can improve the taste, digestibility and nutritional properties. Kaur and Prasad (2021) reported that the concentration of antinutritional factors in chickpea by pre-processing significantly reduced. There are generally three pre-processing methods for chickpea, which were the physical (milling and soaking), biochemical (germination and fermentation) and thermal treatments (roasting and extrusion), respectively (Kaur & Prasad, 2021).

Germination is a conventional and non-thermal process, which embryos control growth. The process of germination of seed takes place in three steps: firstly, the seed is soaked in water, which will absorb water quickly; secondly, the biochemical process activation happens in moisture conditions and the speed of seed water absorption will also slow down; thirdly, the radicle of a seed appears, namely the germination of seed occurs (Wolny et al., 2018). Chemical composition after seed germination was altered, such as the digestibility of protein in chickpea after gemination was 2 times higher than that of the raw chickpea (Milán-Noris et al., 2018); the contents of polyphenols and flavonoids significantly increased (Gupta et al., 2017). Thus it can be seen that the germination processing plays an important role in the improvement of the bioactivity, nutritional qualities, functional and antioxidant properties of chickpea, and it also can decrease the antinutrient factors such as phytic acid, tannins, and α-galacto-oligosaccharides (Wolny et al., 2018, Milán-Noris et al., 2018). At the same time, some studies have also found that the functional properties also were different after the germination of different sorghum varieties (Afify Ael, El-Beltagi, El-Salam, & Omran, 2012). It implies that different varieties of seeds have different nutritional components and functions after germination (Afify Ael et al., 2012).

Although the germination processing of chickpea seed is beneficial to the preparation of flour and its application as a food ingredient in the development of functional food, these studies focus on nutritional composition, physicochemical, starch characteristics, thermodynamic, and functional properties of single chickpea (Kaur & Prasad, 2021). Therefore, the objective of this study was to compare the effect of germination on the compositions, bioactivity, thermal properties, protein secondary structure, volatile and functional characteristics of three varieties of chickpeas. This would establish a basis for understanding the behavior of germinated chickpea in food matrices.

## 2 Materials and methods

### 2.1 Materials

Three varieties of chickpea, namely Kabuli type: Muying1 and Y2-514; Desi type: YZ-364, were collected from Xinjiang Agricultural Sciences. Bicinchoninic acid (BCA) protein assay kit was purchased from USA Pierce Company (Rockford, IL, USA). 1,1-Dipheny-2-picrylhydrazyl (DPPH) and 2,2′-azinobis-(3-ethylbenzthiazoline-6-sulfonate) (ABTS) was bought from Sigma–Aldrich (St. Louis, MO, USA). The pepsin (3000 U/mg) and pancreatin from porcine pancreas (4000 U/mg) were bought from the Novozymes (Bagsværd, Denmark). All other chemicals and solvents used were of analytical grade.

### 2.2 Germination of chickpea seeds and preparation of germinated flour

Germination rate of chickpea seeds: the surface of chickpea seeds was made free from live bacteria or other microorganisms by 2 v/v % sodium hypochlorite solutions for 10 min with occasional stirring and rinsed completely with deionized water (Liu, Childs, Loos, Taylor, Smart, & Abbaspourrad, 2023). The sterilized seeds were soaked in deionized water at ambient temperature (25 °C) for 10–15 h. After washing with water, 50 soaked seeds were evenly spread on four-layer wet gauze, then covered with two layers of gauze, and germinated at 25 °C and 80 % relative humidity in the dark for 72 h. The number of germinated seeds can be obtained by counting. The germination rate was calculated by the following equation:

(1) $$\mathrm{Germinationrate}\left(\%\right)=\frac{\mathrm{Numberofgerminatedseeds}}{\mathrm{Totalnumberofseeds}}\times 100$$

The cleaned raw (directly harvested chickpea) and germinated chickpea seeds were dried at 50 °C in oven until the content of moisture was less than 10 %. Germinated and raw (control) seeds were ground in high speed crusher, passed through 80 mesh sieves to the flour of uniform particle size and stored at 4 °C for the further experiments.

### 2.3 Proximate analysis of germinated and untreated chickpea seed

The contents of moisture, crude protein, ash and vitamin C of the samples were measured by the methods 925.09, 992.23, 923.03 and 967.21 a of AOAC (2005), respectively.

### 2.4 Thermal properties

The thermal properties of the chickpea samples were evaluated by differential scanning calorimetry (DSC, TA Q20, New Castle, USA). DSC was determined according to a previously reported method (Di et al., 2022). 3 mg of samples and 12 μL distilled water were mixed and sealed in aluminum pans. The pan was loaded into the equipment chamber, and then heated in DSC from 20 to 125 °C with a flow rate of 5 °C /min. The empty pan was as the blank (Di et al., 2022). The onset temperature(T_0_), peak temperature(T_p_), conclusion temperature (T_c_) and enthalpy change (△H) was obtained from the thermogram. (Di et al., 2022).

### 2.5 Scanning electron microscopy (SEM)

The morphological properties of raw and germinated chickpeas were estimated by SEM (SU8100, Hitachi High- Technologies Corporation, Tokyo, Japan). The sample flour was fixed on aluminium stub using the double-sided carbon tape, and then coated with a 10 nm gold film by spraying (Sharma, Jan, Riar, & Bansal, 2021). The SEM images were collected at an accelerating voltage of 10 kV and 3000 × magnification.

### 2.6 Fourier transform infrared (FTIR) measurement

The conformation of chickpea flour was investigated by the described method previously (Xing et al., 2023). Briefly, the sample flour was blended with potassium bromide in a ratio of 1:100, and then ground and prepared into a pellet for scanning. The FTIR spectra data of raw and germinated samples were collected by a scanning range of 4000–400 cm^−1^ with a FTIR Spectrometer ((Bruker Daltonics, Bremen, Germany) at a resolution 4 cm^−1^ and averaging 32 scans. The proportion of the protein secondary structures was analyzed by the Origin Pro. 2021 software (Hearne Scientific Software).

### 2.6 Techno-functional properties

Water holding capacity (WHC) and oil holding capacity (OHC) were measured according to the method described by Choe Osorno, Ohm, Chen, & Rao (2022).

The foaming capacity (FC) and foam stability (FS) were determined as described by Di et al. (2022). The protein solution with a concentration of 10 mg/mL was homogenized at 10,000 rpm for 3 min by a homogenizer (PT-10-35GT, BUCHI Labortechnik AG, Switzerland) at 25 °C, and the volume was recorded directly. Then, the volumes of the samples were recorded after maintaining for 30 s. FC was expressed by the following Eq. (1).

(2) $$\mathrm{FC}\left(\%\right)=\frac{\left(V_{1}+V_{2}\right)-V_{0}}{V_{0}}\times 100$$

where V_0_ was the total volume of the liquid before homogenizing, mL; V_1_ was the volume of the foam, mL; V_2_ was the volume of the lower liquid, mL.

The samples were kept for 30 min at ambient temperature, the volume of the foam was recorded again, and FS was expressed the following the equation (2).

(3) $$\mathrm{FS}\left(\%\right)=\frac{\mathrm{Volumeafterkeepingofthefoam}(mL)}{\mathrm{Volumebeforekeepingofthefoam}(mL)}\times 100$$

Emulsification capacity (EC) and emulsion stability (ES) were determined according to a previously reported method (Di et al., 2022). Firstly, soybean oil and sample solution (80 mg/mL) were mixed in a 1:1 ratio. The dispersions was whipped by a high-speed shearing blender (PT-10-35GT, BUCHI Labortechnik AGBUCHI Labortechnik AG, Switzerland) at 10, 000 rpm for 2 min at room temperature. Then, the homogeneous mixture was centrifuged at 2000 × g for 5 min at room temperature. The emulsion solution was allowed to maintain a few minutes until the emulsion layer was steady. The height of the emulsified layer and the total height of the liquid in the tube were registered. EC was calculated as follows:

(4) $$\mathrm{EC}\left(\%\right)=\frac{\mathrm{Heightofemulsifiedlayer}}{\mathrm{Totalheightoftheliquid}}\times 100$$

The above emulsion was centrifugated again, after heating at 80 °C for 30 min and subsequently was cooled at ambient temperature. Then, the samples were centrifuged again for 5 min, and the height of the emulsified layer and the total height of the liquid in the tube were registered. ES was expressed by the following equation:

(5) $$\mathrm{ES}\left(\%\right)=\frac{\mathrm{Heightofemulsifiedlayerafterheating}}{\mathrm{Totalheightoftheliquidafterheating}}\times 100$$

### 2.7 Determination of antioxidant properties

Total phenolic content (TPC) were extracted from un-germinated and germinated chickpea flours according to the method of Chinma et al. (2022) with small modification. In brief, the solid (chickpea flour) and liquid (80 % aqueous methanol) according to the ratio of 1:10 (w/mL) was mixed and kept stirring for 2 h at ambient temperature. The supernatant after centrifugation was filtered solution by a filter paper (Whatman no. 1) and the volume was fixed to 20 mL. The TPC determination of the samples was done using the Folin-Ciocalteu method. 2 mL of Folin-Ciocalteu reagent diluted 10 times, 0.4 mL of extract and 5 mL of distilled water was blended, which was placed to stand in the dark for 5 min, then 3 mL Na_2_CO_3_ (10 % concentration) solution was added. The mixture solution was incubated for 30 min in dark at room temperature and the absorbance was determined at 630 nm using a spectrophotometer (UV-2401PC, Shimadzu Corporation, Kyoto, Japan). 80 % methanol solution was as blank control. the reagent blank TPC content was accounted using gallic acid standard curve (y = 8.6795x + 0.0036, R^2^ = 0.9992) and expressed as mg gallic acid equivalents per 100 g of extract (mg GAE/100 g). All data were showed in triplicate.

Total flavonoids content (TFC) were determined by the NaNO_2_-Al(NO_3_)_3_-NaOH method (Liu et al., 2022). In brief, 2.0 mL of the extract solution and 0.3 mL of 5 % NaNO_2_ was mixed. After reacting at room temperature for 6 min, 0.3 mL of 10 % Al(NO_3_)_3_ and 2.0 mL of 4 % NaOH was added in mixture solution and included for 15 min at room temperature, and the total volume was adjusted to 10 mL using distilled water. The absorbance was measured at 510 nm using a spectrophotometer (UV-2401PC, Shimadzu Corporation, Kyoto, Japan). 80 % methanol solution was as blank control. TFC was calculated by rutin standard curve (y = 0.2127x + 0.0036, R^2^ = 0.9993) and expressed as mg rutin equivalents per g of extract (mg RE/g). All experiments were done in triplicate.

1, 1-Diphenyl-2- picrylhydrazyl (DPPH) radical scavenging activity was performed according to the reported method of Kömürcü and Bilgiçli (2023) with some modification. In brief, 0.5 mL of extract solutions was mixed with 4 mL 0.1 mmol/L methanolic DPPH solution. The absorbance of solution was recorded by a spectrophotometer (UV-2401PC, Shimadzu Corporation, Kyoto, Japan) at 517 nm after keeping for 30 min in the dark. 80 % methanol solution was as a blank control. All experiments were performed in triplicate.

Percentage DPPH scavenging activity was accounted by the equation number (6):

(6) $$\mathrm{DPPHradicalscavenging}\left(\%\right)=\frac{A_{0}-A_{1}}{A_{0}}\times 100$$

where A_0_ was the absorbance of control and A_1_ was the absorbance of sample.

2,2′-azino-bis (3-ethylbenzthiazoline-6-sulphonic acid (ABTS) free radical scavenging ability was assayed by the method of Chinma et al. (2022). Briefly, the ABTS radical was prepared by mixing 7 mmol/L ABTS aqueous solution and 2.45 mmol/L K_2_S_2_O_8_ in 1:1 ratio, which was reacted for 16 h in the dark. The ABTS solution was diluted with 80 % methanol until the absorbance was 0.70 ± 0.02 at 734 nm. 4 mL of the ABTS was mixed with 0.50 mL extract and incubated 10 min at 30 °C in darkness. The absorbance was recorded at 734 nm by a spectrophotometer (UV-2401PC, Shimadzu Corporation, Kyoto, Japan). 80 % methanol was taken as the blank control. All experiments were done in triplicate.

Percentage ABTS scavenging activity of the sample was calculated by the following equation (7).

(7) $$\mathrm{ABTSradicalscavenging}\left(\%\right)=\frac{A_{0}-A_{1}}{A_{0}}\times 100$$

where A_0_ and A_1_ represented the absorbance of control and sample, respectively.

The ferric reducing antioxidant power (FRAP) analysis of extract solution was done according to the method of Kömürcü and Bilgiçli (2023). Briefly, 0.12 mL of the extract was mixed with 3.61 mL of ferric-TPTZ (2,4,6-tripyridyl-s-triazine) reagent. After 30 min in the dark at 37 °C, the absorbance was measured at 593 nm in a spectrophotometer (UV-2401PC, Shimadzu Corporation, Kyoto, Japan). The mixture of 3 mL FRAP reagent and 0.1 mL distilled water was as the blank control. FRAP value was calculated by Trolox standard curve (y = 0.3482x + 0.0103, R^2^ = 0.9915) and expressed as mg Trolox equivalents per mL of extract (mg Trolox/mL). All experiments were done in triplicate.

### 2.8 In vitro protein digestibility (IVPD) of chickpea flours

The IVPD of the raw and germinated chickpea flours was carried out according to the method of Di et al. (2022). Briefly, 2.0 g of chickpea flour and 100 mL 0.1 mol/L sodium citrate tribasic dihydrate (pH 2.5) with pepsin (2.0 g pepsin/L) was mixed in a 250 mL Erlenmeyer flask. The mixture solution was incubated at 37.5 °C for 0, 5, 10, 30, 60 and 120 min in a water bath to simulate gastric digestion. Subsequently, the intestinal digestion was started by adding10 mL of simulated intestinal solution (SIF, including 2000 U of pancreatin and 0.32 g of fresh bile) and the pH value was adjusted to 7.5 using 1 mol/L NaOH. The mixture was incubated at 37.5 °C for 0, 5, 10, 30, 60 and 120 min in a water bath to simulate intestinal digestion. 4 mL of each intestinal phase were taken and heated in a boiling water bath for 10 min to terminate the reaction of enzymes. Then, the sample solution was centrifuged at 5500 rpm for 10 min at ambient temperature, and the protein content in supernatant was determined by the BCA method (Smith et al., 1985). The IVPD of sample was accounted by the following equation (8).

(8) $$\mathrm{IVPD}\left(\%\right)=\frac{{\mathrm{PC}}_{0}-{\mathrm{PC}}_{T}}{{\mathrm{PC}}_{0}}\times 100$$

where PC_0_ represented the initial protein content; PC_t_ represented the protein content after simulated in vitro digestion.

### 2.9 GC-IMS analysis

The volatile organic compounds of raw and un-germinated chickpea were determined by GC-IMS (FlavourSpec®, Dortmund, Germany) according to the method of Li et al. (2023) with slight modifications. Briefly, two grams of accurately weighed chickpea flours were put in a 20 mL of sealed headspace glass sampling vial. After incubating at 55 °C for 15 min, 200 μL of samples were injected (85 °C) into an HS-GC-IMS system by a heated gas-tight syringe for further experiment.

The separation samples were obtained by MXT-5 capillary chromatographic column (15 mL of length; 0.53 mm of internal diameter; 1 μm of film thickness; Restek Corp., Bellefonte, PA, USA) with ultrapure nitrogen (99.99 %) as the carrier. The program design for the nitrogen flow rate was as follows: 2 mL/min for 2 min, 2 mL/min to 10 mL/min for 2–10 min, 10 mL/min to 100 mL/min for10–30 min, 150 mL/ min for 20–30 min. The resulting ions were introduced into a drift tube (9.8 cm in length), which the operated temperature and voltage was 45 °C and 6.5 keV, respectively (Li et al., 2023). The flow rate of drift gas was 150 mL/min. The IMS ionization source (β-rays) was ^3^H (300 MBq) (Li et al., 2023). There was an average of 12 scans per spectrum (Li et al., 2023). The data were analyzed using the NIST (https://chemdata.nist.gov) database via the LAV 2.2.1 software (GAS Dortmund, Dortmund, Germany) (Li et al., 2023). The experiment was performed in three times.

### 2.10 Statistical analysis

All experiments were taken over at least in triplicate. The data in tables and figures were expressed by the mean values ± standard deviation of three times (mean ± SD). The results were analyzed with one-way analysis of variance (ANOVA) and the significant differences were performed with Duncan’s test (*p* < 0.05) using SPSS statistics version 20.0 software (IBM Corporation, Chicago, IL, USA).

## 3 Results and discussion

### 3.1 Growth index of germination and chemical characteristics of different varieties of chickpea flour

One of the important indicators of seed quality is germination rate (Qin et al., 2021). Table 1 shows statistically significant differences between germination chickpea seeds (*p* < 0.05) regarding germination percentage (%) and shoot. The germination percentage and shoot length in three chickpeas was from the range of 31.14 % to 86.94 % and 1.43 to 2.54 cm, respectively. There was the significant difference among the three varieties (p < 0.05). The shoot length and germination rate of YZ-364 with black seed coat were significantly lower than those of other white varieties (p < 0.05). The highest germination rate was Muying1, which reached 86.94 % and more than twice the germination rate of YZ-364. While Muying1 had the longest average shoot length, about 2.54 cm. The results showed that the growth trend of Muying1 and Y2-514 with white coat was fairly good. It was probably attributed that the different varieties of chickpea had different seed sizes and pattern (Coradzzo, 2002). The shape index and weight of Muying1, Y2-514 and YZ-364 were 1.51, 1.45, 1.23 and 4.95, 4.34, 3.61 g, respectively. It was found that the shape index and weight of Muying1 and Y2-514 were higher than YZ-364. Generally, the embryo of larger individual seeds had more nutrients and the water absorption capacity was better than that of small seeds, which would contribute to the gemmation of the chickpea (Akinyosoye, Adetumbi, Amusa, Olowolafe, & Olasoji, 2014).

**Table 1:** Growth index of germination and proximal composition of chickpeas before and after germination.

	Growth index		Proximal chemical composition			
	Germination rate (%)	Shoot Length (cm)	Moisture (%)	Protein (%)	Ash (%)	Vitamin C (mg/100 g)
Germinated samples						
Muying1	86.94 ± 0.71^a^	2.54 ± 0.15^a^	28.66 ± 8.78^ab^	25.92 ± 0.04^d^	2.36 ± 0.04^d^	1261.72 ± 5.3^a^
Y2-514	81.17 ± 1.56^b^	2.12 ± 0.25^ab^	36.44 ± 7.95^a^	26.95 ± 0.05^a^	3.06 ± 0.03^b^	1052.70 ± 4.40^b^
YZ-364	31.14 ± 2.34^c^	1.43 ± 0.03^c^	42.78 ± 3.27^a^	26.27 ± 0.01^ab^	2.59 ± 0.09 ^cd^	381.63 ± 3.11^c^

Raw samples						
Muying1	–	–	4.83 ± 0.52^b^	23.12 ± 1.11^c^	2.66 ± 0.03^c^	66.54 ± 1.72^e^
Y2-514	–	–	5.53 ± 0.66^b^	25.08 ± 0.05^ab^	3.42 ± 0.07^a^	89.55 ± 1.86^d^
YZ-364	–	–	5.80 ± 0.89^b^	24.04 ± 0.02^bc^	3.52 ± 0.09^a^	58.02 ± 0.55^e^

The data of experiments were expressed by the Mean ± SD (n = 3) standard deviation values. For growth index and proximal chemical composition data, the different superscript letters within the same column were significantly different (*p* < 0.05) according to Duncan’s Multiple Range Test.

The moisture, protein, ash and Vitamin C contents of the chickpea seed and sprout flours significantly (*p* < 0.05) varied (Table 1). There was the significant increase for the moisture content after germination, which was probably related to the water absorption of seeds during the germination process. It was attributed that the seeds underwent various metabolism (Megat Rusydi & Azrina, 2012). After germination, the protein content in chickpea seed exhibited an increasing trend. Sofi et al. (2023) also reported that the protein content for germinated desi chickpea increased, which most probably attributed to the emergence of new protein during germination. Ash content had not significant differences before and after germinated chickpea.

The effect of germination on the vitamin C contents of three chickpeas is showed in Table 1. The content of vitamin C in chickpea seeds was lower, though the germination resulted in rapid and gradual increase for three chickpea cultivars. The results in three chickpea cultivars showed that the germination would bring out a significant rise in vitamin C. Because the enzymes involved in metabolism of seeds were generated or activated during germination, which made the metabolism of internal substances in the seed continuously strengthen, and the enzymes involved in vitamin C synthesis and metabolism was also enhanced (Martinez-Villaluenga et al., 2010).

### 3.2 Analysis of thermal properties

Table 2 presents the significant changes in the thermal behavior of the different varieties of chickpea flours after germination (*p* < 0.05). As shown in Table 2, onset (T_0_), peak (Tp), and conclusion (Tc) temperatures decreased significantly (*p* < 0.05). This phenomenon was attributed to the reduction of the fat layer around the starch particles after germination, which would cause the starch particles to expand more easily during the heat treatment process (Sofi et al., 2023). The difference in thermal properties of three chickpea cultivars was due to the different characteristics of the starch particles and different ground substances around them (Sofi et al., 2023). It was also found that the germination significantly reduced the enthalpy of the chickpea flours (Table 2), which might be due to hydrolysis of some starch particles during germination (Sofi et al., 2023). The crystallinity quality and overall crystallinity of the starch in raw materials were evaluated by the peak temperature and enthalpy, respectively (Sofi et al., 2023), and thermal properties could be speculated the gelatinization of the starch crystalline part. In this study, the results suggested that the germination chickpea possessed the lower crystallinity and molecular order than that of raw, which was supported by the study of Sofi et al. (2023). A gradual reduction of ΔH values of the chickpea after germination could be attributed to the fact that the starch had more double-helical order and fewer longer-branch chains (Tang et al., 2021). For example, the raw Muying1 showed the higher enthalpy compared to the raw Y2-514 and YZ-364. The difference in ΔH values could be attributed to differences in the botanical sources and/or three-dimensional structure of their starches (Tang et al., 2021).

**Table 2:** The physical, thermal, functional and antioxidant characteristics of chickpeas before and after germination.

Variety	Muying1		Y2-514		YZ-364	
Treatment	Raw	Germinated	Raw	Germinated	Raw	Germinated
T_0_ (°C)	134.72 ± 1.08^c^	118.89 ± 1.23^e^	136.91 ± 1.15^b^	127.37 ± 0.93^d^	138.47 ± 1.22^a^	136.65 ± 1.37^b^
T_p_ (°C)	137.81 ± 1.42^b^	120.20 ± 2.01^d^	131.35 ± 1.03^c^	121.62 ± 1.36^d^	140.62 ± 1.34^a^	138.38 ± 1.21^b^
T_c_(°C)	142.53 ± 1.27^b^	126.15 ± 1.64^d^	135.97 ± 1.06^c^	124.94 ± 1.18^d^	144.79 ± 1.13^a^	142.16 ± 1.01^b^
ΔH (J/g)	59.56 ± 0.51^a^	34.42 ± 0.54^c^	46.74 ± 0.63^b^	23.13 ± 0.45^f^	29.73 ± 0.38^d^	24.54 ± 0.81^e^
WHC (g/g)	1.96 ± 0.20^d^	4.70 ± 0.17^b^	4.34 ± 0.01^c^	5.43 ± 0.22^a^	4.61 ± 0.12^b^	4.03 ± 0.32^c^
OHC (g/g)	0.70 ± 0.06^b^	0.75 ± 0.11^a^	0.71 ± 0.05^b^	0.72 ± 0.01^b^	0.63 ± 0.10^c^	0.74 ± 0.08^a^
EA (%)	44.23 ± 1.33^b^	58.41 ± 2.18^a^	40.72 ± 1.88^c^	56.49 ± 2.32^a^	45.08 ± 2.17^b^	54.34 ± 1.74^a^
ES (%)	38.35 ± 2.76 ^cd^	43.08 ± 1.57^b^	53.15 ± 2.98^ab^	56.31 ± 2.83^a^	40.92 ± 2.07^c^	56.10 ± 4.82^a^
FC (%)	37.55 ± 1.00^c^	46.17 ± 0.14^a^	36.44 ± 0.17^c^	36.22 ± 0.16^c^	36.18 ± 0.11^c^	40.68 ± 0.81^b^
FS (%)	74.71 ± 1.74^b^	52.10 ± 0.55^e^	58.93 ± 1.86^d^	46.38 ± 1.42^f^	91.56 ± 3.70^a^	63.17 ± 2.06^c^
TPC (mg GAE)/100 g	0.81 ± 0.05^d^	1.37 ± 0.05^c^	0.86 ± 0.03^d^	1.47 ± 0.06^b^	0.88 ± 0.05^d^	1.73 ± 0.12^a^
TFC (mg RE)/g	0.24 ± 0.02^c^	0.32 ± 0.01^b^	0.34 ± 0.01^b^	0.64 ± 0.02^a^	0.33 ± 0.02^b^	0.62 ± 0.02^a^
DPPH activity (%)	35.00 ± 0.01^f^	94.00 ± 0.02^a^	56.90 ± 0.01^c^	79.30 ± 0.01^b^	50.90 ± 0.01^e^	54.80 ± 0.01^d^
ABTS activity (%)	82.31 ± 0.01^d^	97.11 ± 0.01^b^	93.23 ± 0.01^c^	97.43 ± 0.01^b^	94.72 ± 0.04^c^	99.10 ± 0.01^a^
FRAP (mg/mL)	1.17 ± 0.01^e^	1.46 ± 0.01^b^	1.24 ± 0.04^d^	1.35 ± 0.02^c^	1.26 ± 0.02^d^	1.56 ± 0.01^a^

GAE: gallic acid equivalents; RE: rutin equivalents; T_0_, onset temperature; T_p_, peak temperature; T_c_, conclusion temperatures; ΔH, enthalpy; WHC, water-holding capacity; OHC, oil-holding capacity; EA, emulsion activity; ES, emulsion stability; FC, foaming capacity; FS, foam stability; TPC: Total phenolic content; TFC: Total flavonoids content; DPPH: 1, 1-Diphenyl-2- picrylhydrazyl; ABTS: 2,2′-azino-bis (3-ethylbenzthiazoline-6-sulphonic acid; FRAP: ferric reducing antioxidant power. From T_0_ to FRAP values, the different superscript letters in the same row were significantly different (*p <* 0.05) according to Duncan’s Multiple Range Test.

### 3.3 Scanning electron microscopy (SEM) analysis

**Fig. 1:**
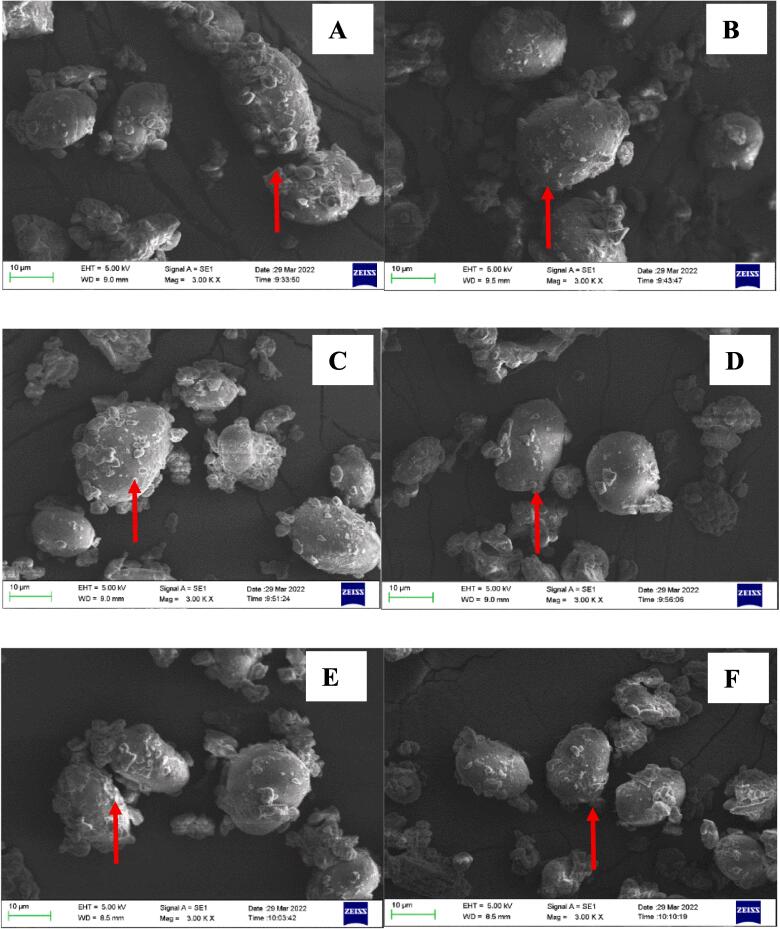
Scanning electron micrographs of un-germinated and germinated chickpea flour. A, C and E, the SEM images (3.0 k × magnification) of Muying1, Y2-514 and YZ-364 seeds without germination treatment, respectively; B, D and F, the SEM images (3.0 k × magnification) of Muying1, Y2-514 and YZ-364 with germination treatment.

SEM was applied to determine the effect of germination on the microstructure of chickpea. The surface morphology of chickpea flours before and after germination is shown in Fig. 1. Micrographs of the raw Muying1, Y2-514 and YZ-364 (Fig. 1 A, C and E) revealed that the starch granule was densely set in fiber and protein matrix. From Fig. 1 B, D and F, it was obviously observed that the granules had slightly disorientated and rearranged in germinated sample. After germination, the structure of protein and fiber matrix grew loose and some starch granules were released from the matrix. These results were consistent with the previously reported changes in the surface morphology of yellow pea and faba bean with germination treatment (Setia, Dai, Nickerson, Sopiwnyk, Malcolmson, & Ai, 2019). During germination, the chickpea seeds took in the water and swelled, which could result in the disruption of the matrix structure (Setia et al., 2019). After drying at 50 °C, the compact structure of chickpea seeds could not be renovated. The matrix of three chickpea after germination was loosen, which caused that the more starch granules were set free as from the structure (Setia et al., 2019). The activated enzymes corresponding to protein and cellulose would destroy their structures in the process of germination, finally, the main structure of chickpeas would also collapse (Setia et al., 2019).

### 3.4 Fourier transform infrared (FTIR) analysis

**Fig. 2:**
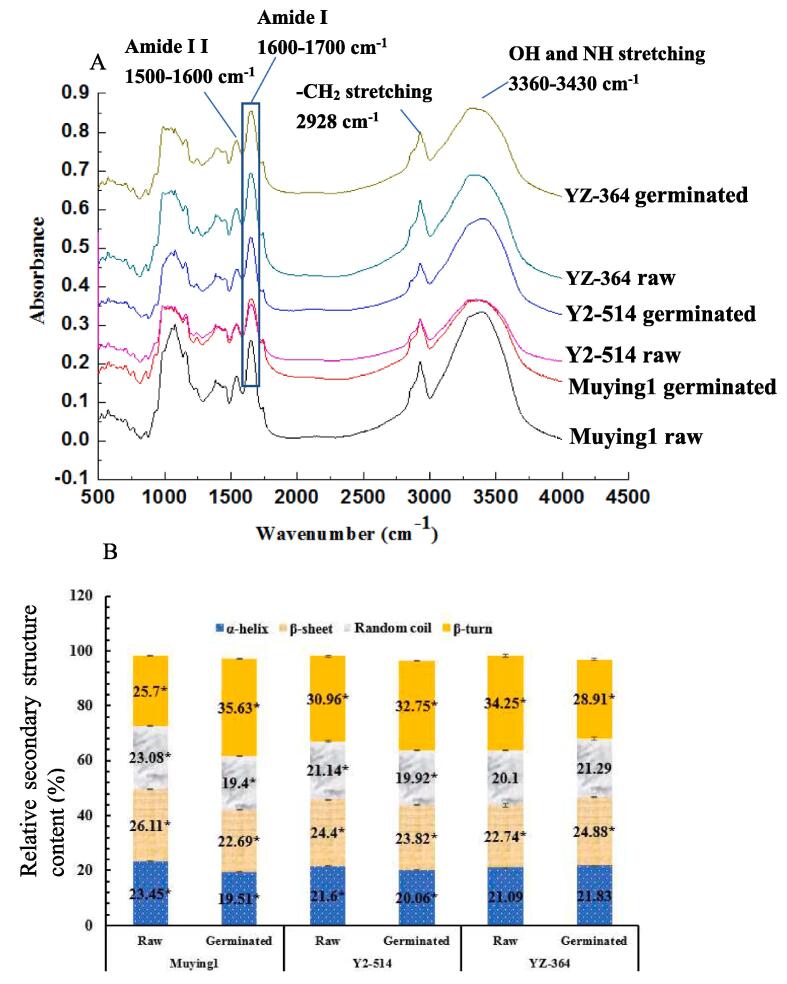

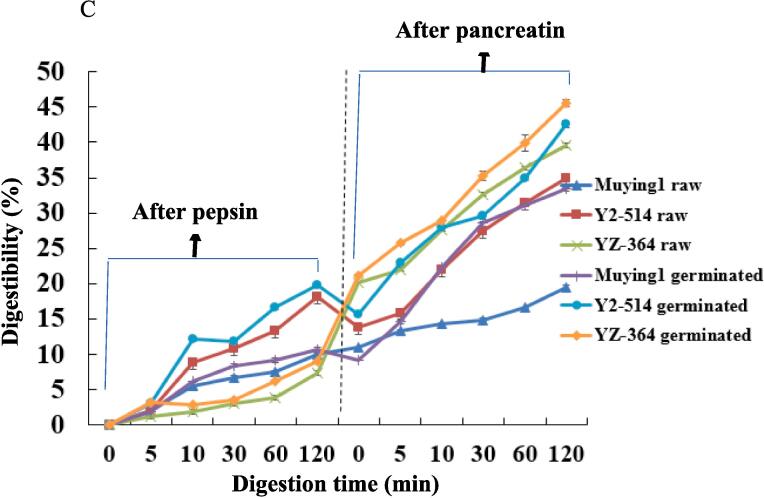
A: Fourier transform infrared spectroscopy (FTIR) results; B: the secondary structure contents of raw and germinated chickpea flours, a star (*) indicated a significant difference (*p <* 0.05) between raw and germinated samples; C: the vitro protein digestibility (IVPD) changes of raw and germinated chickpea flours.

FTIR spectra of raw and germinated three chickpeas in the region between 4000 and 400 cm^−1^ are given in Fig. 2 A, which can reflect the changes of some functional groups. The chickpea flours before and after germination showed a wide band peak 3360 and 3430 cm^−1^ (Fig. 2 A), that it was attributed to the symmetric stretching vibration of O — H and N — H groups from starches, phenolic compounds and proteins of samples (Djordjević, Maravić, Teofilović, Šoronja-Simović, & Šereš, 2023). The intensities of O — H and N — H stretching peaks in chickpea flours increased after germination, which likely could be related to the increase of hydrophilic substance contents during germination (Djordjević et al., 2023). It was observed the occurring the peaks at 2928 cm^−1^ (Fig. 2 A), which were due to asymmetric and symmetric stretching of − CH_2_ groups with being relative to lipids. Their intensities of chickpea flours after germination increased, the reason likely was that the activity of hydrolytic lipase was stronger during germination (Djordjević et al., 2023). However, this result was different from Sofi et al. (2023), which found that the intensities of peaks at 2929 to 2923 cm^−1^ decreased in germinated desi chickpea (GNG-469 and GNG-1581) flour. This indicated that the hydrolysis of lipid in the process of germination was distinct in different chickpea cultivars.

FTIR spectra at 1100–––1700 cm^−1^ corresponds to the chemical groups of protein (Yakubu, Sharma, Sharma, & Singh, 2022). The bands at 1700–1600 cm^−1^ in amide I were attributed to the C=O and C=N group stretching vibration of peptide bond, while the bands at 1575–1480 cm^−1^ in amide II were assigned to the stretching vibration of N — H and C — N groups, which were closely related to the secondary structure of proteins (Djordjević et al., 2023, Yakubu et al., 2022). The increase of peak intensities at 1652, 1540 and 1385 cm^−1^ for three chickpea flours after germination could be relative to the increase of protein content, which was consistent with the *desi* chickpea reported by Sofi et al. (2023). Other reason likely was that the proteins and polypeptides during germination were degraded, which caused the increase of the synthesis of amino acids (Chinma et al., 2022). The peak observed at band in nearby region of 1052 cm^−1^ was attributed to the C — O stretching vibration of carbohydrates, which occurred in un-germinated and germinated chickpea seeds (Dong et al., 2023). As shown in Fig. 2 A, it was found that the different varieties of chickpea showed different peak intensity under the same germination conditions. The intensity of the peak in Muying1 decreased in germination treatment, which could be due to the hydrolysis of the starch by amylases (Sofi et al., 2023). However, it was interesting that the peak intensity in Y2-514 and YZ-364 after germination increased, which was in line with the results previous studies by Dong et al. (2023), they found that the intensity at 1056 cm^−1^ in germinated flaxseed for 4 d increased compared to the raw, indicating that the germination could affect the starch crystallinity structure of Y2-514 and YZ-364. These results also indicated that the different chickpea varieties had different starch hydrolysis, which were probably attributed to the different enzyme activity (Qin et al., 2021). There was an increase of peak intensity at the nearby region of 574 cm^−1^ during germination, which was attributed that the sugar concentration increased with the degradation of complex carbohydrates (Chinma et al., 2022). This change was in agreement with the findings of Chinma et al. (2022), who reported that the peak intensity at around 573 cm^−1^ for pigeon pea increased after germination. Atudorei, Stroe, and Codiná (2021) also found that the changes in peak intensity of these representative spectra were mainly caused by changes in protein, carbohydrate and fat of lentil, soybean, and chickpea.

One major peak of all samples in Amide II regions at around 1544 cm-1 was found. It was possible to observe that raw chickpeas exhibited lower peaks in this spectral region while germination chickpeas had a higher intensity (Fig. 2A), which was related to protein molecular structures (Djordjević et al., 2023, Yakubu et al., 2022). Because the Amide I region had the ‘purest‘ contribution from changes in secondary structure of proteins and was the one that undergoes the largest changes, it was chosen for further analysis (Vaskoska et al., 2021). The amide I in FTIR spectra mainly contained the secondary structure (α-helix, β-sheets, random coil unordered and β-turn) of proteins (Yakubu et al., 2022), including α-helix at 1650–1660 cm^−1^, β-sheet at 1610–1642 cm^−1^, random coil at 1642–1650 cm^−1^ and β-turn at 1660–1700 cm^−1^. The secondary structure contents of chickpea flours before and after germination are shown in Fig. 2 B. From the Fig. 2 B, the main constituents of the protein secondary structure in un-germinated and germinated Muying1, Y2-514 and YZ-364 were the β-turn and β-sheet proportion, while the random coil proportion was the lowest. After germination, the proportion of α-helix, β-sheet and random coil in Muying1 and Y2-514 significantly decreased (*p* < 0.05), while that of β-turn significantly increased (*p* < 0.05) (Di et al., 2022). The contents of α-helix and random coil in YZ-364 before and after germination were not significantly different (*p* > 0.05), β-sheet and β- turn decreased by 2.14 % and 5.34 %, respectively. This result was inconsistent with the change of secondary structure in germinated Muying1 and Y2-514. These results indicated that the germination process could affect the structure of protein in different kinds of chickpea (Sofi et al., 2023).

### 3.5 Functional properties of chickpea flours

There were significant changes in the functional properties of the raw and germinated chickpea flour cultivars (Table 2). Water holding capacity (WHC) of germinated Muying1, Y2-514 and YZ-364 significantly increased (*p* < 0.05). it was probably attributed that the modified macromolecules during germination processing caused the increase of WHC from the germinated flours, such as the starch polymer structures were changed to the monomers (Chinma, Abu, Asikwe, Sunday, & Adebo, 2021). The result indicated that the germination would be helpful to improve the functionality of chickpea products with high hydration property (Chinma et al., 2021). Oil holding capacity (OHC) of raw Muying1, Y2-514 and YZ-364 was 0.70, 0.71 and 0.63 g/g, respectively, while these values in germinated flours increased to 0.75, 0.72 and 0.74 g/g, respectively, which might be due to the favorable absorption of hydrophobic proteins and fats (Du, Jiang, Yu, & Jane, 2014). This result was agreement with the finding of Sofi et al. (2023), who reported an increase in OHC in germinated desi chickpea.

The emulsion capacity (EC) and stability (ES) of raw Muying1, Y2-514 and YZ-364 seeds were 44.23 and 38.35 %, 40.72 and 53.15 %, 45.08 and 40.92 %, respectively. After germination, EC and ES of chickpea flours significantly increased (*p* < 0.05). It was supposed that the position of hydrophobic amino acids was exposed with the appearance of dissociation and partial unfolding of polypeptide chain during germination, which would constitute the compounds with lipid droplets, and then produced the higher EC (Chinma et al., 2021). The increase of ES was ascribed that the germination could be helpful to form the strong electrostatic repulsive force on the surface of oil droplets in the oil water emulsion, which would lead to the upper stability (Mesfin, Abera Belay, & Endale Amare, 2021). The results of EC and ES were in line with Mesfin et al. (2021).

The foaming capacity (FC) values of raw Muying1, Y2-514 and YZ-364 seeds were 37.55, 36.44 and 36.18 %, respectively. Through comparison to raw samples, it was found that the FC in germinated Muying1 and YZ-364 significantly increased to 46.17 % and 40.68 % (*p* < 0.05), respectively, while the FC in raw and germinated Y2-514 was similar. The increase behavior was due to the increase of protein solubility after chickpea germination (Chinma et al., 2021). It was noting that when the volume of foam increases, the stability of foam decreases. The foaming stability (FS) values of raw Muying1, Y2-514 and YZ-364 were 74.71 %, 58.93 % and 91.56 %, respectively. Un-germinated YZ-364 with the lowest FC was the highest FS. After germination, these values significantly reduced to 52.10, 46.38 and 63.17 % (*p* < 0.05), respectively. It was ascribed that the proteolytic enzymes acted in protein molecules during germination, which caused the change of configuration of proteins and peptides (Chinma et al., 2021).

The functionality of germinated chickpea flour indicated that the germination as low-cost and traditional processing method with no chemical modification or using a chemical additive would improve the functional properties of chickpea flour (Chinma et al., 2021).

### 3.6 Effects of germination on antioxidant activity

Total phenolic and flavonoids content (TPC and TFC) of the chickpea flours was showed in Table 2. TPC of raw Muying1, Y2-514 and YZ-364 was 0.81, 0.86 and 0.88 mg GAE/100 g, respectively. Germination significantly increased the TPC of Muying1, Y2-514 and YZ-364 (p < 0.05), which was 1.37, 1.47 and 1.73 mg GAE/100 g, respectively. It likely was attributed to the activation of endogenous hydrolase (such as phenylalanine ammonia lyase, a key enzyme in phenol biosynthesis) in chickpea seeds (Sharma et al., 2021). This finding was in line with the study of Mesfin et al. (2021). Chen, Zhu, and Qin (2022) also reported that the germination could cause the increase of the phenolic compound contents in buckwheat.

It was also found that YZ-364 was the variety with the highest TFC before and after germination. TFC of raw Muying1, Y2-514 and YZ-364 was 0.24, 0.34 and 0.33 mg RE/g, respectively. Germination treatment increased TFC content for three varieties of chickpea seeds. It was probably as following: (1) the flavonoid enzymes (such as chalcone isomerase) promoted the biosynthesis; (2) other secondary plant metabolites caused by seed coat germination; (3) the cotyledons produced by enzyme activation (Duodu, 2014).

As shown in Table 2, the data indicated that the total antioxidant of chickpea flours significantly was affected by the germination (*p* < 0.05). After germination of chickpea, DPPH radical scavenging activity of Muying1, Y2-514 and YZ-364 (p < 0.05), increased significantly from 35.00 to 94.00 %, 56.90 to 79.30 % and 50.90 to 54.80 %, respectively (Table 2) (*p* < 0.05). The results disclosed that the increase of DPPH radical scavenging activity of germinated chickpea might be attributed to the higher TPC content (Sharma et al., 2021). The finding was supported by the previous studies (Mesfin et al., 2021).

ABTS radical scavenging activity of raw Muying1, Y2-514 and YZ-364 was 82.31, 93.23 and 94.72 %, respectively. Germination significantly improved ABTS scavenging capacity for three varieties compared to their raw counterparts. The increase of antioxidant capacity during germination might be due to the release of bound phenolics from the seed coats (Mesfin et al., 2021). In addition, there was a correlation between changes in the secondary structure of proteins and the antioxidant activity of samples (Sheoran et al., 2013). When the total antioxidant capacity and the ability to scavenge ABTS free radicals showed an upward trend, the secondary structure of proteins β-turn also increased correspondingly (Fig. 2 B) (Sheoran et al., 2013). In this study, it was found that the β- turn content in germinated YZ-364 increased the most, which increased from 25.70 % to 35.63 % (Fig. 2 B). The increase of ABTS percentage in germinated YZ-364 was also the highest, which reached 99.10 %.

FRAP value of the raw Muying1, Y2-514 and YZ-364 was 1.17, 1.24 and 1.26 mg/ml, respectively. The FRAP values in three chickpea cultivars increased significantly during germination (*p* < 0.05). The increment of 24.79, 8.87 and 23.81 % was observed in three chickpeas, respectively (Table 2). The maximum increase was obtained for germinated YZ-364, which was due to the higher phenolic content (Chen et al., 2022).

From the above results, it can be obtained that there was a correlation between phenols and antioxidant activity for the raw and germinated chickpea seeds.

### 3.7 In vitro protein digestibility (IVPD) of raw and germinated chickpea fours

The digestibility of protein largely determines its nutritional quality (Dong, Wang, & Raghavan, 2021). In general, the large proteins must be broken down into small peptides or amino acids to pass through the small intestine wall, and these nutrients eventually enter the blood (Dong et al., 2021). The influence of germination treatment on the IVPD of chickpea seeds is given evidence in Fig. 2 C. After pepsin digestion, the digestibility of the germinated Muying1, Y2-514 and YZ-364 increased to varying degrees than that of un-germinated samples, while the growth rate of Muying 1 was the largest (Fig. 2C). After pancreatin digestion, the significant increase of IVPD was observed in the germinated samples (*p* < 0.05). The IVPD of Muying1, Y2-514 and YZ-364 protein increased from 19.42 to 33.41 %, 34.97 to 42.52 % and 39.55 to 45.62 %, respectively. This phenomenon occurred due to the partial hydrolysis of protein during seed germination (Di et al., 2022). Thereby, the content of water-soluble protein and free amino acids in the whole chickpea powder increased, which could cause the increase of protein IVPD (Di et al., 2022). At the same time, the intrinsic protease of seeds during germination processing was stimulated, which could lead to the increase of IVPD (Di et al., 2022). Some references also reported that the IVPD of yellow pea, faba bean and Bambara ground nut after germination increased (Chinma et al., 2021, Setia et al., 2019). By this study, it suggested that the germination could improve IVPD up to a point, which was in accordance with results of Setia et al. (2019).

As a key feature of protein application, IVPD affects the absorption amount of protein in the human body. The high IVPD protein is considered high-quality proteins, it is attributed that the hydrolysis of protein can promote the release of amino acids from protein skeleton, which indicates that the body can better digest and absorb these nutrients (Mir, Riar, & Singh, 2020). Thus, the results of this study proved that the use rate of protein in chickpea could be risen by germination, which would be helpful to improving the application in food industry.

### 3.8.Volatile Compounds of chickpea flour identified by HS-GC-IMS

**Fig.3:**
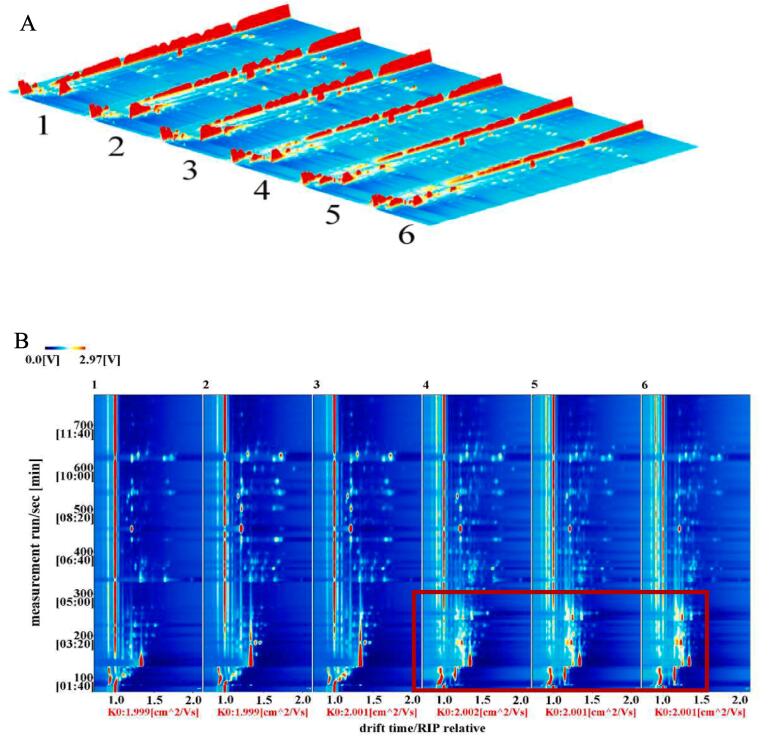

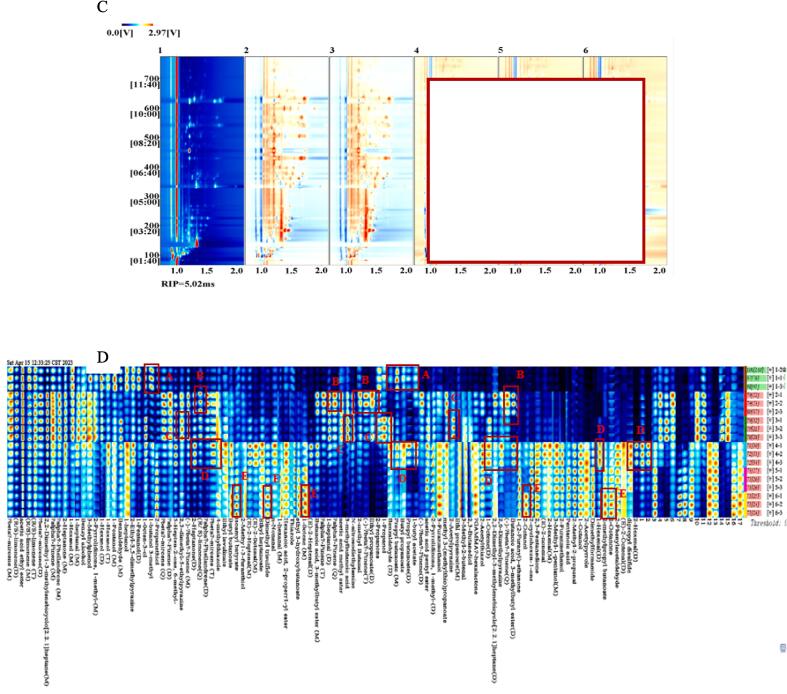
GC-IMS topography of the volatile compounds of raw and germinated chickpea flours. (A) Volatile matter composition spectrum (top view); (B) three-dimensional spectrum of volatile matter composition; (C) comparative difference spectrum of volatile matter composition; (D) the fingerprint of volatile compounds in raw and germinated chickpea flours. 1, 2 and 3 presented raw Muying1, Y2-514 and YZ-364 flours, respectively; 4, 5 and 6 presented germinated Muying1, Y2-514 and YZ-364 flours, respectively.

The volatile compounds in chickpea flours before and after germination were analyzed by HS-GC-IMS. Fig. 3 A presented a typical 3D topography of chickpea samples. As shown in the 3D topographic plot, the peak signal intensity of chickpea flours before and after germination showed the similar visualizations, but the signal intensity presented some differences. When the chickpea seeds were germinated, the contents of volatile compounds exhibited different changes.

The different volatile profiles of raw and germinated chickpea flour samples are further distinguished by the top view of the GC-IMS 3D topographic plot due to the relatively rough 3D spectrum, which was shown in Fig. 3B (Xuan et al., 2022). The abscissa and the ordinate of the spectrum was the drift time and the retention time of GC, respectively. The total headspace compounds of raw and germinated chickpea flour were presented by the whole spectrum. Each point on the right of the reaction ion peak (RIP) corresponds to a volatile compound, the larger the point, the darker the color, indicating a stronger peak signal (Xuan et al., 2022). From Fig. 3B, it was observed that there were most of signals in the drift time of 1.0–1.5 and the retention time of 100–600 s. The raw Muying1 was selected as a reference because of the differences in volatile compound content and the difficulty of comparing them, and the other samples were discounted by the reference. The difference comparison model was used to compare the raw and germinated chickpea flours (Fig. 3B). In Fig. 3B, the red presented the high intensity of each identified compound, while the blue hinted low intensity (Yang et al., 2021). As shown in Fig. 3B, the differences of volatile compounds among raw and germinated chickpea flours were found in the position and intensity of the ion peaks. For germinated chickpea flours, the number of red dots increased obviously compared to raw chickpea flours, indicating that the concentrations of volatile substances in germinated chickpea flours were significantly higher than those of the raw samples at the drift time 1.0–1.5 (*p* < 0.05) (Fig. 3B).

Although the topographic plots visually can display the change trend of flavor components in sprouted chickpea flours. It is difficult to correctly draw conclusions about individual volatile compounds on the topographic map. The different volatile compound fingerprints and dynamic changes in each compound of different samples were automatically identified by Gallery plug-in Plot, in order to interpret the changes of volatiles in chickpea seeds after germination. The signal intensity of volatile compounds was reflected by the color degree of points. The brighter color represents a higher content, while the darker color represented lower content (Yang et al., 2021). In addition, the increase, decrease, absence, or fluctuation changes of volatile compounds between different samples can be contrasted and declared by fingerprints. As shown in Fig. 3C, the fingerprinting spectrum is a very effective method for understanding the details of each flavor compound. All the determined volatile compounds were spread in different regions. The fingerprint profile order of six different chickpea flours from top to bottom was raw Muying1, raw Y2-514, raw YZ-364, germinated Muying1, germinated Y2-514 and germinated YZ-364. To clarify the changes in volatiles during chickpea germination, all peaks of the fingerprint were automatically generated and analyzed for samples by the Gallery Plot plug-in (Fig. 3D). As shown in Fig. 3D, the fingerprint presented the complete volatile information of each sample, and the differences between raw and germinated treatment samples were obvious. The gallery plots of samples were separated into A, B, C, D and E region, which represented the peak intensity differences of volatile compound between raw and germination treatment chickpea samples (Fig. 3D). The raw Muying1 characteristic peak area (A region) mainly contained propyl butanoate(M), butyl propanoate and 3-methyl-1-butanol. The characteristic peak area (B region) of raw Y2-514 mainly contained butanoic acid 3-methylbutyl ester(D), (-)-beta-pinene(Q), 2,2-dimethyl-3-methylenebicyclo[2.2.1] heptane(M) and (-)-beta-pinene(D). The raw YZ-364 characteristic peak area (C region) mainly contained 2-acetylpyrazine, 1-propanol, *N*-nitrosodiethylamine, 3-methylbutanoic acid and 2,3-dimethyl-5-ethylpyrazine. The characteristic peak area of germinated Muying1 (D region) mainly contained dipropyl disulfide, (E)-2-octenal(D), 1-hexanal(D), 2-hexenal(D), (E) −2-nonenal, butanoic acid 3-methylbutyl ester(D), 2,6-dimethylpyrazine, 1-octene(D), methyl 3-(methylthio)propanoate, 1-butyl acetate, propyl butanoate(D), butyl propanoate, propyl butanoate(M), benzaldehyde(D), (E)-2-heptenal(D), ethyl heptanoate, (E)-2-octenal(D), (E)-2-heptenal(M), beta-mircene(T) and 2-heptanone(D). The characteristic peak area of germinated YZ-364 (E region) mainly contained 2-octanone, 2-methylpropyl butanoate, 2-octanol, 1-octene(M), ethyl 3-hydroxybutanoate, dimethyl trisulfide and isoamyl butyrate. These results exhibited that the combination, contents, and intersection of volatile compounds appeared the differences between raw and germinated chickpea samples, the content of some volatile components significantly increased after germinating chickpea seeds (*p* < 0.05), such as 1-(2-Furanyl)-ethanone, 2-cyclohexen-1-one, 2,3-Pentanedione, Ethyl propanoate(M) et al., while some compounds significantly decreased (*p* < 0.05), such as Butyl propanoate, beta-mircene(M), (R/S)-limonene(M), Phellandrene(M) and acetic acid ethyl ester et al.

Table 3 show the detailed information of identified volatile compounds, which revealed that the germinated Muying1, Y2-514 and YZ-364 had a different volatile emission compared to its corresponding raw seed. 103 volatile compounds were identified, which generally represented all types of aroma-producing substances in chickpea (Table 3), containing 22 terpenes, with higher contents of beta-mircene, limonene and 2,2-Dimethyl-3-methylenebicyclo[2.2.1]heptane; 18 aldehydes, with the higher contents of 2-methyl butanal, 3-methyl-2-butenal, benzaldehyde, (E)-2-heptenal and 1-heptanal; 19 esters, with highest content of acetic acid ethyl ester; 17 alcohols, with the higher contents of 2,3-butanediol, 3-methyl-1-butanol, 1-hexanol and 1-pentanol; 10 ketones, with the higher contents of 5-hepten-2-one, 6-methyl-, 2-propanone and 2-heptanone; 1 furan, with the content of 1-(2-Furanyl)-ethanone; 16 others, with the higher contents of thiazole, 3-methylbutanoic acid, dimethylformamide and 2-propanethiol. The concentration of these volatile compounds changed because of different varieties of chickpea and germination treatment. Though the most volatile compounds in all chickpea samples were terpenes, the contents of acetic acid ethyl ester and 2,2-Dimethyl-3-methylenebicyclo[2.2.1]heptane (M) were higher.

**Table 3:** Identified volatile compounds of raw and germinated chickpea flour based on GC-IMS.

Volatiles	Compound	Count	CAS#	Formula	MW	RI	Rt [*sec*]	Dt [a.u.]	Intensity (V)					
									1	2	3	4	5	6
Terpenes	beta-mircene(M)	6	C123353	C_10_H_16_	136.20	995.70	545.19	1.22	1548.02 ± 22.42^c^	1709.41 ± 17.91^b^	1735.02 ± 6.65^a^	1222.06 ± 40.96^d^	1095.44 ± 18.46^e^	989.32 ± 13.94^f^
	beta-mircene(D)	7	C123353	C_10_H_16_	136.20	994.90	543.50	1.29	841.36 ± 18.36^c^	1112.68 ± 39.33^a^	1049.97 ± 13.09^b^	762.46 ± 29.8^d^	560.74 ± 4.15^e^	433.6 ± 6.48^f^
	beta-mircene(T)	8	C123353	C_10_H_16_	136.20	994.70	543.08	1.64	228.03 ± 3.47^d^	527.54 ± 12.56^a^	465.27 ± 13.5^b^	441.52 ± 13.64^c^	211.47 ± 6.49^d^	178.22 ± 2.21^e^
	beta-mircene(Q)	9	C123353	C_10_H_16_	136.20	994.70	543.08	1.71	889.41 ± 26.08^c^	1842.58 ± 79.8^a^	1654.13 ± 43.07^v^	903.85 ± 30.96^c^	703.81 ± 8.5^d^	549.73 ± 10.56^e^
	(R/S)-limonene(M)	14	C138863	C_10_H_16_	136.20	1038.90	623.98	1.21	3714.48 ± 85.25^a^	3141.59 ± 104.74^b^	3230.24 ± 33.00^b^	3068.56 ± 85.25^bc^	2956.28 ± 19.9^c^	2786.21 ± 13.53^d^
	(R/S)-limonene(D)	15	C138863	C_10_H_16_	136.20	1042.60	631.15	1.30	3225.61 ± 23.12^b^	4253.48 ± 52.63^a^	4332.99 ± 7.51^a^	3336.66 ± 69.01^b^	2648.55 ± 44.13^c^	2625.12 ± 66.43^c^
	(R/S)-limonene(T)	16	C138863	C_10_H_16_	136.20	1038.60	623.38	1.64	2143.6 ± 49.94^b^	2268.8 ± 6.06^b^	2354.45 ± 3.31^a^	2051.55 ± 63.28^b^	1784.43 ± 16.12^c^	1532.86 ± 12.88^d^
	(R/S)-limonene(Q)	17	C138863	C_10_H_16_	136.20	1040.50	626.97	1.72	2094.03 ± 28.27^e^	4792.69 ± 81.25^a^	4177.96 ± 66.63^b^	3289.83 ± 56.31^c^	2262.12 ± 49.66^d^	2047.97 ± 75.86^e^
	alpha-Phellandrene(M)	10	C99832	C_10_H_16_	136.20	1009.40	569.24	1.21	1296.51 ± 41.35^d^	2191.75 ± 45.85^a^	2084.12 ± 23.88^b^	1503.66 ± 64.15^c^	1191.22 ± 26.45^e^	1022.02 ± 19.32^f^
	alpha-Phellandrene(D)	11	C99832	C_10_H_16_	136.20	1009.40	569.24	1.68	581.34 ± 19.89^d^	1946.4 ± 173.09^a^	1500.89 ± 56.06^b^	953.07 ± 59.81^c^	551.87 ± 15.56^d^	415.08 ± 13.66^e^
	(-)-beta-Pinene(M)	22	C127913	C_10_H_16_	136.20	975.90	505.03	1.22	2011.96 ± 79.95^d^	4885.51 ± 163.64^a^	4279.02 ± 57.47^b^	2786.33 ± 161.52^c^	1812.97 ± 65.75^e^	1597.42 ± 32.7^f^
	(-)-beta-Pinene(D)	23	C127913	C_10_H_16_	136.20	978.50	510.10	1.30	95.52 ± 3.96^d^	379.11 ± 33.02^a^	259.8 ± 7.53^b^	128.59 ± 10.90^c^	60.23 ± 2.39^f^	67.02 ± 0.70^e^
	(-)-beta-Pinene(T)	24	C127913	C_10_H_16_	136.20	975.50	504.36	1.64	79.08 ± 8.14^e^	798.64 ± 94.16^a^	497.49 ± 13.89^b^	276.83 ± 17.46^c^	100.34 ± 5.01^d^	72.46 ± 1.41^e^
	(-)-beta-Pinene(Q)	25	C127913	C_10_H_16_	136.20	976.30	505.88	1.73	43.66 ± 1.58^d^	366.6 ± 44.96^a^	227.78 ± 4.37^b^	119.04 ± 6.69^c^	48.16 ± 4.61^d^	42.08 ± 1.72^d^
	2,2-Dimethyl-3-methylenebicyclo[2.2.1]heptane(M)	31	C79925	C_10_H_16_	136.20	950.20	457.29	1.22	6270.86 ± 188.42^d^	13601.21 ± 396.13^a^	12254.35 ± 91.36^b^	9159.61 ± 373.65^c^	6279.5 ± 171.08^d^	5606.92 ± 91.41^d^
	alpha-Pinene(M)	33	C80568	C_10_H_16_	136.20	934.40	430.16	1.22	1291.51 ± 43.18^d^	1754.79 ± 60.8^b^	1901.33 ± 6.49^a^	1481.33 ± 56.61^c^	1165.3 ± 26.22^e^	994.14 ± 15.81^f^
	alpha-Pinene(D)	90	C80568	C_10_H_16_	136.20	934.60	430.35	1.30	400.61 ± 9.93^d^	876.65 ± 37.74^a^	778.59 ± 12.67^b^	556.51 ± 31.74^c^	373.74 ± 7.25^e^	383.93 ± 10.34^e^
	alpha-Pinene(T)	91	C80568	C_10_H_16_	136.20	934.50	430.33	1.67	367.84 ± 34.54^f^	2682.59 ± 331.63^a^	1893.48 ± 41.61^b^	1195.55 ± 85.86^c^	542.39 ± 34.95^d^	439.39 ± 12.92^e^
	alpha-Pinene(Q)	92	C80568	C_10_H_16_	136.20	934.80	430.71	1.72	91.08 ± 5.75^f^	885.41 ± 104.52^a^	631.27 ± 13.49^b^	365.51 ± 26.1^c^	153.68 ± 11.55^d^	135.94 ± 4.41^e^
	1-Octene(D)	109	C111660	C_8_H_16_	112.20	790.20	254.33	1.45	78.04 ± 7.56^e^	170.64 ± 12.76^d^	375.25 ± 13.2^a^	312.74 ± 6.93^b^	267.87 ± 17.1^c^	257.41 ± 9.49^c^
	1-Octene(M)	110	C111660	C_8_H_16_	112.20	797.60	261.06	1.16	407.79 ± 11.27^f^	468.2 ± 33.09^e^	535.95 ± 13.12^d^	912.55 ± 36.33^c^	1297.74 ± 24.27^b^	1416.12 ± 9.01^a^
	2,2-Dimethyl-3-methylenebicyclo[2.2.1]heptane(D)	119	C79925	C_10_H_16_	136.20	949.00	455.16	1.74	173.57 ± 11.34^d^	446.54 ± 64.13^a^	286.55 ± 2.16^b^	279.3 ± 8.69^b^	242.55 ± 3.71^c^	253.49 ± 3.83^c^
Aldehydes	(E) −2-nonenal	1	C18829566	C_9_H_16_O	140.20	1144.80	866.73	1.41	90.84 ± 1.89	78.34 ± 2.10	107.55 ± 0.92	269.84 ± 1.37	153.99 ± 5.37	121.62 ± 4.13
	2-methyl Butanal	65	C96173	C_5_H_10_O	86.10	650.00	156.56	1.41	426.31 ± 34.11^e^	926.51 ± 211.93^a^	785.75 ± 45.34^b^	685.38 ± 37.61^c^	516.65 ± 30.6^d^	296.5 ± 10.92^f^
	2-Methyl-2-propenal	83	C78853	C_4_H_6_O	70.10	568.40	126.17	1.20	437.88 ± 20.77^e^	399.73 ± 4.39^e^	308.63 ± 10.08^f^	1493.93 ± 31.87^c^	1630.15 ± 7.61^b^	1666.72 ± 4.24^a^
	3-Methyl-2-butenal	93	C107868	C_5_H_8_O	84.10	789.30	253.49	1.35	226.1 ± 17.58^e^	758.75 ± 42.95^c^	932.24 ± 21.17^a^	820.78 ± 22.65^b^	836.58 ± 9.54^b^	652.87 ± 14.33^d^
	*n*-Nonanal	111	C124196	C_9_H_18_O	142.20	1103.20	761.78	1.47	107.15 ± 3.39^c^	107.56 ± 2.6^c^	167.94 ± 7.03^b^	186.8 ± 13.52^a^	108.79 ± 0.21^c^	96.96 ± 3.52^d^
	2-Phenylacetaldehyde	115	C122781	C_8_H_8_O	120.20	1053.50	652.84	1.25	63.49 ± 4.23^e^	52.32 ± 2.10^f^	78.73 ± 2.89^d^	357.24 ± 3.13^a^	328.08 ± 3.46^b^	310.12 ± 1.81^c^
	(E)-2-Octenal(M)	2	C2548870	C_8_H_14_O	126.20	1067.50	681.91	1.34	67.96 ± 2.04^e^	56.22 ± 2.55^f^	109.5 ± 4.75^c^	252.45 ± 3.43^a^	146.86 ± 1.52^b^	98.53 ± 1.55^d^
	Benzaldehyde(M)	26	C100527	C_7_H_6_O	106.10	962.70	479.80	1.15	583.54 ± 76.60^b^	374.36 ± 8.38^c^	737.78 ± 23.66^a^	599.57 ± 34.49^b^	576.92 ± 6.22^b^	593.1 ± 18.75^b^
	Benzaldehyde(D)	27	C100527	C_7_H_6_O	106.10	962.50	479.47	1.47	48.95 ± 6.60^c^	27.77 ± 1.26^d^	90 ± 6.97^a^	70.71 ± 11.41^b^	45.34 ± 0.43^c^	40.78 ± 3.90^c^
	(E)-2-Heptenal(M)	29	C18829555	C_7_H_12_O	112.20	957.90	471.03	1.26	398.96 ± 22.73^e^	314.57 ± 19.03^e^	565.03 ± 15.55^b^	822.12 ± 37.70^a^	559.43 ± 11.3^b^	450.33 ± 6.05^c^
	(E)-2-Heptenal(D)	30	C18829555	C_7_H_12_O	112.20	958.20	471.52	1.67	82.31 ± 8.28^e^	69.94 ± 6.22^f^	167.78 ± 5.99^c^	426.09 ± 41.77^a^	190.06 ± 4.42^b^	125.85 ± 5.85^d^
	1-heptanal(M)	37	C111717	C_7_H_14_O	114.20	900.90	377.75	1.35	506.81 ± 9.5^c^	685.12 ± 24.4^a^	714.47 ± 20.64^a^	574.23 ± 28.21^b^	426.87 ± 5.94^d^	332.3 ± 5.48^e^
	1-heptanal(D)	38	C111717	C_7_H_14_O	114.20	900.90	377.75	1.69	194.3 ± 9.35^d^	643.45 ± 9.90^a^	621.13 ± 24.93^a^	411.71 ± 31.54^b^	214.86 ± 2.26^c^	180.01 ± 4.99^d^
	2-Hexenal(M)	48	C505577	C_6_H_10_O	98.10	848.40	312.67	1.18	50.19 ± 4.15^d^	97.83 ± 9.68^c^	105.92 ± 1.29^c^	277.83 ± 18.55^a^	168.64 ± 10.68^b^	178.73 ± 10.15^b^
	2-Hexenal(D)	49	C505577	C_6_H_10_O	98.1	848.30	312.53	1.52	14.89 ± 0.54^f^	25.35 ± 5.13^e^	44.04 ± 0.9^d^	514.18 ± 35.93^a^	163.14 ± 7.33^b^	66.21 ± 2.39^c^
	1-hexanal(M)	53	C66251	C_6_H_12_O	100.20	797.70	261.14	1.25	462.24 ± 17.86^d^	495.75 ± 24.55^d^	433.47 ± 2.28^e^	613.22 ± 32.16^c^	822.68 ± 19.36^b^	891.6 ± 11.18^a^
	1-hexanal(D)	54	C66251	C_6_H_12_O	100.20	791.00	255.08	1.56	27.6 ± 1.52^e^	16.33 ± 0.70^f^	110.21 ± 8.71^d^	380.01 ± 6.47^a^	231.45 ± 10.73^c^	282.85 ± 6.35^b^
	(E)-2-Octenal(D)	113	C2548870	C_8_H_14_O	126.20	1067.70	682.15	1.82	10.55 ± 0.67^d^	10.74 ± 0.93^d^	12.58 ± 0.57^c^	36.68 ± 1.46^a^	18.55 ± 0.98^b^	13.06 ± 0.33^c^
Esters	Butyl butanoate	19	C109217	C_8_H_16_O_2_	144.20	1000.40	553.66	1.35	324.43 ± 2.67^f^	464.55 ± 10.74^d^	400.5 ± 8.42^e^	658.64 ± 4.46^b^	486.47 ± 10.39^c^	686.1 ± 6.33^a^
	2-methylpropyl butanoate	32	C539902	C_8_H_16_O_2_	144.20	949.80	456.47	1.33	137.22 ± 8.09^f^	216.27 ± 3.95^d^	170.28 ± 8.28^e^	319.58 ± 9.64^c^	368.09 ± 0.84^b^	491.18 ± 4.32^a^
	ethyl 3-hydroxybutanoate	34	C5405414	C_6_H_12_O_3_	132.20	916.30	400.92	1.16	200.74 ± 13.43^f^	377.73 ± 15.88^e^	403.1 ± 9.08^d^	592.77 ± 7.24^c^	831.55 ± 27.39^b^	973.36 ± 30.39^a^
	1-butyl acetate	56	C123864	C_6_H_12_O_2_	116.20	805.20	268.18	1.62	50.15 ± 7.07^b^	52.58 ± 20.52^b^	38.94 ± 1.51	82.41 ± 9.26^a^	17.82 ± 0.7^d^	35.73 ± 1.77^c^
	acetic acid ethyl ester	67	C141786	C_4_H_8_O_2_	88.10	604.50	138.80	1.33	21052.49 ± 63.69^c^	23720.37 ± 35.05^b^	24162.36 ± 77.15^a^	19388.85 ± 292.73^d^	17576.8 ± 196.67^d^	15750.83 ± 204.75^d^
	acetic acid methyl ester	70	C79209	C_3_H_6_O_2_	74.10	530.90	114.28	1.20	318.74 ± 52.15^e^	727.61 ± 49.40^a^	578.68 ± 7.50^b^	511.73 ± 32.13^c^	428.72 ± 11.34^d^	258.38 ± 2.82^f^
	acetic acid pentyl ester	78	C628637	C_7_H_14_O_2_	130.20	915.80	400.20	1.31	147.17 ± 2.96^d^	548.5 ± 38.8^b^	725.43 ± 34.22^a^	594.91 ± 49.25^b^	544.03 ± 33.5^b^	292.8 ± 22.49^c^
	isoamyl butyrate	88	C106274	C_9_H_18_O_2_	158.20	1044.40	634.60	1.40	729.31 ± 31.67^d^	628.35 ± 12.38^e^	617.48 ± 9.55^e^	943.5 ± 10.21^c^	1066.14 ± 7.62^b^	1237.37 ± 2.79^a^
	Butyl propanoate	95	C590012	C_7_H_14_O_2_	130.20	907.70	387.90	1.29	124.54 ± 7.58^a^	53.16 ± 7.46^c^	65.04 ± 0.62^b^	78.41 ± 10.91^b^	22.03 ± 0.24^e^	44.04 ± 1.88^d^
	Hexanoic acid, 2-propen-1-yl ester	103	C123682	C_9_H_16_O_2_	156.20	1078.20	704.75	1.38	62.38 ± 8.64^d^	44.59 ± 3.42^e^	37.71 ± 0.82^f^	114.35 ± 3.29^a^	91.94 ± 1.39^b^	81.75 ± 2.68^c^
	GAMMA-hexalactone	106	C695067	C_6_H_10_O_2_	114.10	1056.70	659.30	1.53	32.06 ± 6.64^c^	114.42 ± 9.99^a^	94.42 ± 1.41^b^	117.5 ± 2.84^a^	108.63 ± 1.64^a^	103.35 ± 4.32^a^
	Ethyl heptanoate	107	C106309	C_9_H_18_O_2_	158.20	1093.70	739.55	1.41	49.11 ± 0.77^f^	78.13 ± 3.64^e^	116.24 ± 2.76^b^	147.98 ± 2.51^a^	102.65 ± 3.8^c^	82.74 ± 1.67^d^
	methyl 3-(methylthio)propanoate	108	C13532188	C_5_H_10_O_2_S	134.20	1025.60	598.69	1.62	45.89 ± 2.49^e^	265.58 ± 11.24^a^	171.78 ± 13.55^b^	167.36 ± 11.07^b^	109.39 ± 0.30^c^	75.28 ± 2.12^d^
	Ethyl levulinate	120	C539888	C_7_H_12_O_3_	144.20	1063.80	674.13	1.19	53.63 ± 1.18^c^	72.64 ± 1.71^b^	91.98 ± 3.47^a^	94.64 ± 5.86^a^	63.91 ± 0.02^b^	39.66 ± 1.73^d^
	Butanoic acid, 3-methylbutyl ester(D)	12	C109193	C_9_H_18_O_2_	158.20	1026.30	600.05	1.89	30.34 ± 1.46^e^	391.64 ± 41.90^a^	98.41 ± 4.94^c^	329.83 ± 14.03^b^	101.92 ± 4.63^c^	54.77 ± 2.3^d^
	Butanoic acid, 3-methylbutyl ester(M)	13	C109193	C_9_H_18_O_2_	158.20	1025.00	597.52	1.38	218.34 ± 7.83^e^	1283.4 ± 65.68^a^	672.21 ± 22.05^c^	1193.46 ± 32.15^b^	647.11 ± 13.91^c^	381.37 ± 12.96^d^
	Propyl butanoate(M)	40	C105668	C_7_H_14_O_2_	130.20	895.70	370.30	1.26	475.13 ± 35.24^a^	184.47 ± 21.54^d^	224.6 ± 8.49^c^	298.22 ± 16.43^b^	140.2 ± 5.42^e^	229.25 ± 7.34^c^
	Propyl butanoate(D)	41	C105668	C_7_H_14_O_2_	130.20	895.30	369.62	1.68	183.03 ± 30.25^b^	98.61 ± 19.25^c^	118.69 ± 1.85^c^	277.15 ± 44.47^a^	25.54 ± 1.88^e^	76.19 ± 8.46^d^
	Ethyl propanoate(M)	97	C105373	C_5_H_10_O_2_	102.10	705.60	184.86	1.16	507.73 ± 11.37^c^	511.17 ± 52.65^c^	448.12 ± 29.31^d^	1099.41 ± 27.35^b^	1180.03 ± 17.14^a^	1174.42 ± 13.04^a^
Alcohols	1-Octanol	3	C111875	C_8_H_18_O	130.20	1067.70	682.33	1.46	62.34 ± 6.69^d^	51.47 ± 3.45^e^	61.06 ± 0.98^d^	297.14 ± 10.56^a^	270.51 ± 1.38^b^	255.11 ± 4.50^c^
	2-Octanol	18	C123966	C_8_H_18_O	130.20	1001.90	556.20	1.47	55.73 ± 7.13^f^	108.69 ± 8.92^d^	133.02 ± 8.25^c^	207.07 ± 7.13^b^	90.26 ± 1.04^e^	391.79 ± 4.41^a^
	3-Methyl-1-pentanol(M)	50	C589355	C_6_H_14_O	102.20	848.30	312.53	1.31	52.17 ± 2.98^f^	69.40 ± 4.68^e^	107.96 ± 2.67^d^	552.25 ± 3.13^a^	479.55 ± 1.99^b^	409.7 ± 1.49^c^
	2-Furanmethanol	51	C98000	C_5_H_6_O_2_	98.10	848.70	312.97	1.37	12.61 ± 1.77^e^	120.75 ± 4.63^d^	171.57 ± 11.34^c^	612.99 ± 49.6^a^	629.83 ± 5.97^a^	487.58 ± 5.98^b^
	2,3-Butanediol	62	C513859	C_4_H_10_O_2_	90.10	777.10	242.37	1.37	190.59 ± 29.4^f^	812.1 ± 32.46^d^	977.39 ± 41.40^b^	1077.58 ± 97.15^a^	917.04 ± 14.34^c^	669.62 ± 26.13^e^
	3-methyl-1-butanol	63	C123513	C_5_H_12_O	88.10	728.50	201.64	1.50	628.34 ± 21.06^a^	351.09 ± 27.88^c^	315.33 ± 5.46^d^	478.86 ± 23.29^b^	355.44 ± 8.83^c^	319.52 ± 4.09^d^
	1-Propanol	68	C71238	C_3_H_8_O	60.10	566.00	125.39	1.24	223.48 ± 28.22^b^	207.55 ± 12.45^b^	379.91 ± 4.80^a^	165.97 ± 22.36^c^	114.08 ± 3.30^d^	86.86 ± 1.71^e^
	3-Furanmethanol	85	C4412913	C_5_H_6_O_2_	98.10	975.40	504.07	1.35	87.36 ± 10.2_e_	166.17 ± 5.36^c^	134.16 ± 3.07^d^	178.7 ± 4.18^b^	179.93 ± 9.28^b^	196.88 ± 6.03^a^
	1-Octen-3-ol	87	C3391864	C_8_H_16_O	128.20	983.50	520.18	1.16	663.54 ± 8.72^c^	548.26 ± 12.92^d^	1000.54 ± 19.24^a^	906.65 ± 29.86^a^	714.9 ± 18.28^b^	626.57 ± 20.44^c^
	1-heptanol	96	C111706	C_7_H_16_O	116.20	975.90	505.13	1.40	289.46 ± 2.84^a^	149.19 ± 0.72^f^	169.14 ± 3.71^e^	219.49 ± 5.75^b^	197.97 ± 6.40^c^	178.81 ± 3.54^d^
	Benzyl alcohol	98	C100516	C_7_H_8_O	108.10	1045.00	635.76	1.78	190.49 ± 11.58^c^	362.9 ± 6.88^a^	359.19 ± 8.96^a^	234.76 ± 17.11^b^	156.37 ± 2.64^d^	161.61 ± 7.62^d^
	1-Hexanol(M)	46	C111273	C_6_H_14_O	102.20	864.40	330.97	1.33	887.25 ± 6.02^a^	784.81 ± 4.95^c^	849.06 ± 4.41^b^	371.31 ± 9.02^f^	400.3 ± 8.33^e^	416.58 ± 2.67^d^
	1-Hexanol(D)	47	C111273	C_6_H_14_O	102.20	868.90	336.24	1.64	2165.78 ± 101.03^a^	1517.82 ± 14.74^c^	1793.74 ± 16.85^b^	1164.43 ± 38.55^d^	782.62 ± 12.64^e^	614.74 ± 15.19^f^
	1-Hexanol(T)	52	C111273	C_6_H_14_O	102.20	867.50	334.54	1.99	353.62 ± 14.72^a^	229.86 ± 7.86^c^	277.93 ± 3.29^b^	347.49 ± 11.35^a^	216.2 ± 0.51^c^	162.67 ± 6.12^d^
	1-Pentanol(M)	57	C71410	C_5_H_12_O	88.10	759.00	226.34	1.26	966.85 ± 24.06^a^	510.05 ± 17.47^d^	549.58 ± 9.26^c^	547.11 ± 5.01^c^	630.26 ± 3.06^b^	628.2 ± 6.06^b^
	1-Pentanol(D)	58	C71410	C_5_H_12_O	88.10	758.10	225.56	1.51	1049.81 ± 78.65^a^	523.37 ± 36.45^d^	786.17 ± 14.46^b^	662.88 ± 28.18^c^	380.83 ± 7.18^e^	220.96 ± 7.04^f^
	Ethyl propanoate(D)	66	C105373	C_5_H_10_O_2_	102.10	705.20	184.62	1.46	108.83 ± 14.90^e^	1305.76 ± 369.16^a^	1027.49 ± 80.14^b^	271.55 ± 29.50^c^	134.27 ± 23.88^d^	76.95 ± 8.63^f^
Ketones	5-Hepten-2-one, 6-methyl-	21	C110930	C_8_H_14_O	126.20	990.70	534.75	1.18	1165.3 ± 31.75^e^	2386.46 ± 228.43^b^	2446.39 ± 105.81^b^	2790.13 ± 61.71^a^	1748.02 ± 5.3^c^	1407.47 ± 34.99^d^
	2-cyclohexen-1-one	45	C930687	C_6_H_8_O	96.10	888.90	360.97	1.41	158.35 ± 13.15^e^	237.2 ± 15.89^c^	188.33 ± 4.01^d^	1094.96 ± 103.57^b^	1176.21 ± 22.28^a^	1110.5 ± 20.43^b^
	2,3-Pentanedione	64	C600146	C_5_H_8_O_2_	100.10	708.40	186.88	1.23	224.67 ± 13.96^c^	170.27 ± 9.73^d^	215.08 ± 15.03^c^	1255.17 ± 54.56^a^	1284.5 ± 1.85^a^	1141.09 ± 12.23^b^
	2-Propanone	71	C67641	C_3_H_6_O	58.10	511.00	108.41	1.10	1586.28 ± 71.65^a^	1200.1 ± 30.66^c^	1339.47 ± 41.85^b^	623.54 ± 125.21^d^	363.51 ± 4.66^e^	350.78 ± 12.57^e^
	2-Octanone	*86*	*C111137*	*C_8_H_16_O*	*128.20*	*990.30*	*534.00*	*1.32*	*197.44 ± 12.45^d^*	*251.64 ± 3.55^c^*	*183.15 ± 7.33^d^*	*259.98 ± 15.04^c^*	*347.05 ± 13.85^b^*	*432.28 ± 13.11^a^*
	2-Heptanone(M)	42	C110430	C_7_H_14_O	114.20	889.70	362.04	1.27	668.66 ± 16.59^b^	814.56 ± 9.69^a^	851.84 ± 13.19^a^	852 ± 25.84^a^	685.73 ± 10.42^b^	591.85 ± 8.12^c^
	2-Heptanone(D)	43	C110430	C_7_H_14_O	114.20	889.00	361.10	1.63	293.87 ± 2.44^f^	1287.4 ± 24.25^b^	1113.53 ± 37.36^c^	1481.75 ± 82.85^a^	766.13 ± 27.96^d^	389.76 ± 10.73^e^
	2-Pyrrolidinone, 1-methyl-(M)	100	C872504	C_5_H_9_NO	99.10	1056.60	659.20	1.12	162.6 ± 9.49^b^	157.97 ± 6.31^b^	201.74 ± 6.96^a^	105.54 ± 2.98^c^	89.38 ± 0.93^d^	69.68 ± 2.69^e^
	2-Pyrrolidinone, 1-methyl-(D)	101	C872504	C_5_H_9_NO	99.10	1055.90	657.78	1.43	240.83 ± 10.49^e^	452.23 ± 12.22^c^	505.32 ± 7.63^bc^	568.84 ± 4.31^a^	515.11 ± 5.08^b^	437.34 ± 2.14^d^
Furan	1-(2-Furanyl)-ethanone	36	C1192627	C_6_H_6_O_2_	110.10	915.60	399.92	1.45	57.49 ± 4.61^e^	161.71 ± 17.09^c^	113.15 ± 2.83^d^	320.12 ± 2.34^b^	403.83 ± 16.03^a^	349.43 ± 13.48^b^
Others	2-Acetylthiazol	20	C24295032	C_5_H_5_NOS	127.20	1025.50	598.58	1.49	67.07 ± 3.5^f^	560.53 ± 17.1^b^	213.37 ± 6.57	673.63 ± 9.45^a^	529.28 ± 0.26^d^	432.83 ± 17.46^e^
	Thiazole	60	C288471	C_3_H_3_NS	85.10	733.20	205.23	1.27	1215.48 ± 8.14^d^	752.19 ± 14.28^e^	692.29 ± 20.64^f^	1350.56 ± 28.99^c^	1421.69 ± 24.93^b^	1591.25 ± 8.97^a^
	Dimethyl trisulfide	28	C3658808	C_2_H_6_S_3_	126.30	962.40	479.30	1.29	204.84 ± 8.85^d^	168.98 ± 4.8^e^	267.56 ± 18.24^c^	479.01 ± 15.36^bc^	497.51 ± 6.84^b^	597.69 ± 35.14^a^
	Pentanoic acid	35	C109524	C_5_H_10_O_2_	102.10	915.70	400.01	1.23	111.58 ± 4.78^f^	377.12 ± 16.85^d^	307.5 ± 4.11^e^	648.39 ± 33.89^c^	899.14 ± 25.6^a^	799.27 ± 11.68^b^
	3-methylbutanoic acid	79	C503742	C_5_H_10_O_2_	102.10	868.70	336.01	1.49	505.81 ± 55.16^c^	1195.26 ± 22.63^b^	1526 ± 39.16^a^	450.37 ± 43.58^d^	386.39 ± 14.8^e^	300.96 ± 6.13^f^
	*N*-nitrosodiethylamine	39	C55185	C_4_H_10_N_2_O	102.10	900.80	377.61	1.52	99.45 ± 7.23^f^	512.13 ± 4.55^b^	624.86 ± 24.44^a^	247.53 ± 20.53^c^	170.15 ± 8.44^d^	136.18 ± 6.88^e^
	Dimethylformamide	61	C68122	C_3_H_7_NO	73.10	775.00	240.42	1.25	136.56 ± 5.5^d^6	102.68 ± 4.23^e^	98.9 ± 2.69^f^	1347.45 ± 57.22^c^	2171.27 ± 84.46^b^	2499.48 ± 38.21^a^
	2-Propanethiol	69	C75332	C_3_H_8_S	76.20	565.10	125.08	1.15	810.36 ± 65.12^d^	811.65 ± 7.85^d^	800.27 ± 9.85^d^	1306.07 ± 94.87^c^	1513.49 ± 28.88^b^	1706 ± 12.53^a^
	2-Methyl-3-furanthiol	99	C28588741	C_5_H_6_OS	114.20	867.50	334.59	1.14	197.46 ± 2.26^d^	169.45 ± 12.91^e^	163.71 ± 5.61^e^	202.02 ± 5.99^c^	282.84 ± 13.02^b^	343.28 ± 7.59^a^
	2,6-Dimethylpyrazine	77	C108509	C_6_H_8_N_2_	108.10	914.60	398.33	1.54	9.18 ± 0.5e	118.25 ± 13.03^b^	100.77 ± 4.92^b^	132.81 ± 10.91^a^	75.89 ± 2.66^c^	30.36 ± 1.26^d^
	2-Acetylpyrazine	89	C22047252	C_6_H_6_N_2_O	122.10	1024.80	597.17	1.21	138.32 ± 6.47^d^	374.58 ± 18.96^b^	626.21 ± 6.21^a^	362.22 ± 14.04^b^	356.94 ± 8.81^b^	309.7 ± 11.39^c^
	2,3-dimethyl-5-ethylpyrazine	104	C15707343	C_8_H_12_N_2_	136.20	1088.90	728.68	1.22	191.74 ± 1.81^e^	426 ± 10.20^b^	469 ± 8.33^a^	302.96 ± 8.05^c^	251.21 ± 6.69^d^	311.68 ± 7.58^c^
	2-Ethyl-3,5-dimethylpyrazine	121	C13925070	C_8_H_12_N_2_	136.20	1078.60	705.64	1.23	313.5 ± 7.25^a^	232.33 ± 6.2^c^	252 ± 4.04^b^	294.84 ± 13.27^a^	166.57 ± 4.00^d^	110.85 ± 3.92^e^
	4-methylthiazole	94	C693958	C_4_H_5_NS	99.20	805.10	268.09	1.35	234.11 ± 7.58^f^	484.75 ± 28.9^b^	537.7 ± 14.64^a^	299.02 ± 5.97^d^e	251.58 ± 5.64	362 ± 2.57^c^
	3-Methylphenol	102	C108394	C_7_H_8_O	108.10	1088.70	728.21	1.11	129.13 ± 22.08^c^	152.1 ± 4.59^b^	169.29 ± 9.69^a^	121.59 ± 7.91^c^	102.94 ± 2.4^c^	86.7 ± 6.00^d^
	2-Acetylpyrrole	112	C1072839	C_6_H_7_NO	109.10	1051.50	648.76	1.49	6.87 ± 1.21^e^	14.63 ± 1.06^d^	11.17 ± 0.26^d^	142.74 ± 28.69^c^	179.78 ± 10.27^a^	154.63 ± 10.89^b^
	dipropyl disulfide	114	C629196	C_6_H_14_S_2_	150.30	1105.50	767.25	1.26	25.61 ± 1.58^e^	29.99 ± 0.40^e^	38.12 ± 3.24^d^	140.57 ± 1.6^a^	104.08 ± 2.16^b^	50.27 ± 0.75^c^

The data of experiments were expressed by the Mean ± SD (n = 3) standard deviation values. Different superscript letters in the same row were significantly different (*p <* 0.05) according to Duncan’s Multiple Range Test. 1, 2 and 3 presented raw Muying1, Y2-514 and YZ-364 seeds, respectively; 4, 5 and 6 presented germinated Muying1, Y2-514 and YZ-364 seeds, respectively.

As shown in Table 3, after germination treatment, some volatile compounds also gradually increased or decreased, such as the limonene (lemon aroma) contents in germinated Muying1, raw Y2-514, raw YZ-364 significantly decreased (*p* < 0.05), while the hexanal (grassy aroma) increased. The intensity of the “green” and “grassy” flavors of germinated chickpea seeds increased, primarily due to the profit of 1-hexanal (M and D) and 2-hexenal (M and D), which was similar to the reported discovery of germinated wheat (Aung, Kim, Kim, Shin, & Kim, 2023). The 1-propanol,1-hexanol (M, D and T) and 1-pentanol (D) contents in germinated chickpea flour showed a decreasing trend, which might be caused by the esterification of alcohols (Xuan et al., 2022), such as the content of glutamic acid degraded during germinating (Aung et al., 2023). Therefore, the raw chickpea seeds had a strong fruity flavor and burnt taste (Aung et al., 2023). The relative peak area of the 1-(2-furanyl)-ethanone was significantly lower in raw chickpea seeds than in germinated samples, the result was supported by the finding of germinated wheat (Aung et al., 2023). This substance is associated with burnt, acetone and musty. Acetic acid ethyl ester had the highest content in the raw and germinated chickpea seeds, which had an aroma of fruit and gave chickpea seed a pleasant and ethereal fruity (grape) aroma (Yang et al., 2021). The identified ketones included 2-octanone, 2-cyclohexen-1-one, 2,3-pentanedione, 2-propanone and 1-methyl-(M) pyrrolidinone, which added to the fruity, mint, milk aroma and sweet taste of the product, the content of 2-octanone, 2-cyclohexen-1-one, 2,3-pentanedione increased after germinating, on the contrary to 2 propanone and 1-methyl-(M) pyrrolidinone. In addition, the total aldehyde content in the germinated chickpea seeds was higher than that of raw. Compared to the raw chickpea flour, the total contents of terpenes, esters, alcohols and ketones in germinated Muying1 increased, while the contents in germinated Y2-514 and YZ-364 seeds decreased. It was concluded that the germinating had been presented to change the flavor components of the chickpea seeds.

The reasons for the formation of volatile flavor components during germination may be as follows: firstly, the activities of lipoxygenase, hydroperoxide lyase and alcohol dehydrogenase during the formation of volatile substances increased and underwent oxidation reaction, reductive reaction or hydrolysis reaction; secondly, there were the interactions between some aldehydes and amino acids; thirdly, some unsaturated fat acids were degraded (Xuan et al., 2022). These results indicated that the germination could be potential for effects on sensory characteristic of the related chickpea products. Moderate germination not only improves the nutritional structure of chickpeas, but also benefits human consumption, and it can improve the processing performance of chickpeas. The unpleasant green bean odor during chickpea processing will affect the quality of the product, while the germination can provide reference for the production and processing of chickpea products with good flavor. The change in flavor substances of chickpeas after germination treatment will also be beneficial for finding the optimal processing technology.

## 4 Conclusions

In this research, the influences of germination treatment on the structure, functional properties, volatile compounds and in vitro protein digestibility of chickpea cultivars were carried out. The present results showed negligible impacts on the other chemical components in chickpea flours besides of a significant increase in vitamin C content. Germinated chickpea flours had lower transition temperatures values and enthalpy values than that of raw chickpea seeds. In comparison with the corresponding raw chickpea flours, FTIR data showed that the proportion of β-sheet and random coil in germinated YZ-364 chickpea flour increased by 2.14 and 1.19 %, respectively, β-turn decreased from 34.25 % to 28.91 %, while α-helix was similar; the proportion of α-helix, β-sheet and random coil in germinated Y2-514 decreased by 1.54, 0.58 and 1.21 %, respectively, while β-turn increased from 30.96 % to 32.75 %; the proportion of α-helix, β-sheet and random coil in germinated Muying1decreased by 3.95, 3.42 and 3.68 %, respectively, while β-turn increased by 9.93 %. WHC, OHC, EC, ES and FC of germinated chickpea flours flour increased than that of raw chickpea, while FS decreased. The germination promoted an increase in the TPC and TFC, which caused the increase of antioxidant properties. It was attributed that the phenolic compounds during germination could be biosynthesized. The germinated chickpea flours had the higher IVPD, which would be beneficial to improve the availability of chickpeas. 103 of volatile compounds were definitely detected in chickpea flours, which was mainly composed of terpene, aldehydes, ketones, alcohols and others. There were significant differences between contents of volatile compounds of germinated and raw chickpea flours. Therefore, the germination could enhance the nutritional composition, bioactivity, functionality properties and volatile compounds of different chickpea cultivars, which would be beneficial to expand its application in food processing.

## CRediT authorship contribution statement

**Hongyan Mao:** Conceptualization, Software. **Shuo Yuan:** Investigation, Writing – original draft. **Qin Li:** Data curation, Methodology, Writing – original draft. **Xiaoyan Zhao:** Funding acquisition, Project administration, Resources, Supervision, Writing – review & editing. **Xiaowei Zhang:** Data curation, Methodology, Writing – original draft. **Hongkai Liu:** Conceptualization, Software. **Ming Yu:** Formal analysis, Validation. **Meng Wang:** Formal analysis, Validation.

## Declaration of competing interest

The authors declare that they have no known competing financial interests or personal relationships that could have appeared to influence the work reported in this paper.

## Data Availability

Data will be made available on request.
